# Development of a method for screening short-lived proteins using green fluorescent protein

**DOI:** 10.1186/gb-2004-5-10-r81

**Published:** 2004-09-28

**Authors:** Xin Jiang, Philip Coffino, Xianqiang Li

**Affiliations:** 1 Panomics Inc, 2003 East Bayshore Road, Redwood City, CA 94063, USA; 2 University of California, San Francisco, Department of Microbiology and Immunology, San Francisco, CA 94143, USA; 3 Henry Wellcome Laboratories for Integrative Neuroscience and Endocrinology, Bristol University, Whitson Street, Bristol BS1 3NY, UK

## Abstract

We have developed a screening technology for the identification of short-lived proteins. A green fluorescent protein (GFP)-fusion cDNA library was generated for monitoring degradation kinetics. Cells expressing a subset of the GFP-cDNA expression library were screened to recover those in which the fluorescence signal diminished rapidly when protein synthesis was inhibited. Thirty clones that met the screening criteria were characterized individually. Twenty-three (73%) proved to have a half-life of 4 hours or less.

## Abstract 2

A method for identifying short-live proteins using a GFP-fusion cDNA library for monitoring degradation kinetics is described.

## Background

Cellular proteins differ widely in their lability, ranging from those that are completely stable to those with half-lives measured in minutes. Proteins with a short half-life are among the most critical to the cell. Regulated degradation of specific proteins contributes to the control of signal transduction pathways, cell-cycle control, transcription, apoptosis, antigen processing, biological clock control, differentiation and surface receptor desensitization [1,2]. Rapid turnover makes it possible for the cellular level of a protein to change promptly when synthesis is increased or reduced [3]. Furthermore, degradation rate is itself subject to regulation. For instance, inflammatory stimuli cause the rapid degradation of IκBα, the inhibitor of NFκB, resulting in the activation of that transcription factor [4-6].

Analysis of labile proteins has been time-consuming and labor-intensive. The most definitive form of analysis requires pulse-chase labeling cells and immunoprecipitation extracts. *In vitro *assay of degradation is simpler than *in vivo *analysis, but an *in vitro *assay system may not fully mimic the degradation of proteins in the cells. Genome-wide functional screening and systemic characterization of cellular short-lived proteins has received little attention [7]. GFP, the green fluorescent protein from the jellyfish *Aequorea victoria*, has been widely used to monitor gene expression and protein localization [8]. Recently, we demonstrated that fusion of GFP to the degradation domain of ornithine decarboxylase [9], a labile protein, can destabilize GFP [10] and that the degradation of an IκB-GFP fusion protein can be monitored by GFP fluorescence [11]. These studies demonstrate that introducing GFP as a fusion within the context of a rapidly degraded protein does not alter the degradation properties of the parent molecule, and that the GFP moiety of the fusion protein is degraded along with the rest of the protein. GFP fluorescence, which provides a sensitive, rapid, precise and non-destructive assay of protein abundance, can therefore be used to monitor protein degradation [12]. Furthermore, fluorescence associated with single cells can be analyzed using fluorescence-activated cell sorting (FACS), a technology easily adapted to high-throughput screening [13].

We developed a GFP-based, genome-wide screening method for short-lived proteins. We made a GFP fusion expression library of human cDNAs and introduced the library into mammalian cells. Transfected cells were FACS-fractionated into subpopulations of uniform fluorescence. Individual subpopulations were treated with cycloheximide (CHX) to inhibit protein synthesis and re-sorted after 2 hours of treatment. Sorting was gated to recover cells with a fluorescent signal that was diminished compared to the population mode. Repeated application of this process resulted in a high yield of clones that encode labile fusion proteins.

## Results

**Figure 1:**
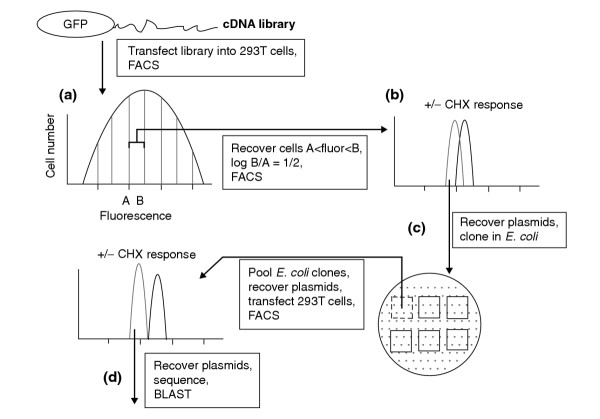
Schematic diagram of the four steps of the screening procedure. **(a) **Fractionate by FACS cells transfected with an EGFP-cDNA expression library according to their fluorescence intensities; **(b) **refractionate those cells made dimmer by cycloheximide (CHX) treatment; **(c) **recover plasmids, clone in bacteria, pool clones and select CHX-responsive pools by FACS analysis; **(d) **recover and characterize individual cDNA clones.

The selection scheme is shown in Figure 1. GFP-cDNA expression libraries were transfected into mammalian cells and cells fractionated into subpopulations, each with a narrow range of fluorescence intensities. Subpopulations were then twice enriched for cells with the desired characteristics. Plasmid DNAs were recovered from the selected cells, subjected to sequence analysis and functionally verified. We made the expression libraries with modified pEGFP C1/C2/C3 vectors by cloning the cDNAs downstream of EGFP. The titer of the library was found to be high: around 10^6 ^cell transformants per microgram of DNA. In addition, we confirmed by PCR amplification that 95% of clones contained a cDNA insert larger than 800 base-pairs (bp) (data not shown). The libraries were thus deemed to be useful for screening short-lived proteins in mammalian cells. We used 293T cells as the recipient. These cells offer two advantages. First, they express the SV40 large T antigen. This allows the library plasmids, which contain an SV40 origin of replication, to be highly replicated. Plasmids can therefore be recovered easily. Second, 293T cells have high transfection efficiency.

**Figure 2:**
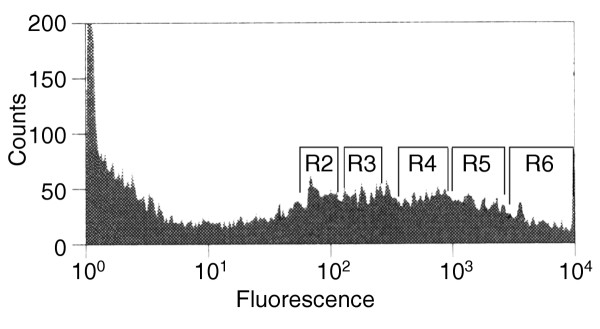
Fractionation of 293T cells transfected with a GFP-cDNA expression library. Cells were subjected to FACS analysis and fractionated into five subpopulations: R2, R3, R4, R5 and R6.

**Figure 3:**
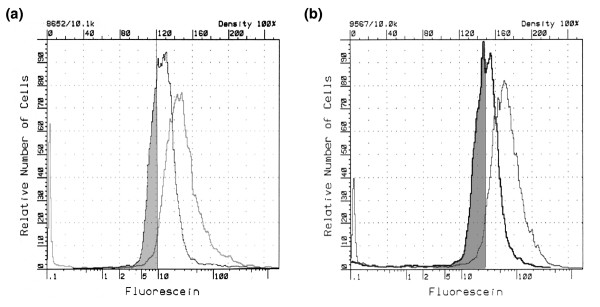
FACS analysis of fractionated cells treated with CHX or untreated. The fractionated subpopulations R3 and R4 treated with or without CHX were subjected to FACS analysis. The log-normal fluorescence histogram distributions from **(a) **R3 and **(b) **R4 populations are shown. The gray curve represents cell populations not treated with CHX and the black curve represents the treated cells. The shaded area represents cells from the populations left-shifted by CHX that were used for plasmid recovery.

After we introduced the GFP-fusion libraries into the mammalian cells, the transfected cells were easily separated by FACS from non-transfected cells or cells transformed by non-productive constructs. We imposed selection for cells that became less bright within 2 hours of exposure to cycloheximide (CHX), a protein synthesis inhibitor. We chose a short treatment time to avoid selecting cells that became dimmer as a result of secondary responses other than rapid turnover of the GFP tagged proteins. To enrich for cells that are susceptible to CHX treatment, we started with a cell population that has an approximately log-normal fluorescence histogram distribution, with a working range of 1.5 to 4.5 logs. We used FACS fractionation to divide this population into five subpopulations (R2, R3, R4, R5, R6) of ascending brightness, gating each on successive one-half log_10 _intervals of fluorescence (Figure 2). Each subpopulation (R2-R6) was divided into two; one portion was treated with 100 μg/ml CHX for 2 hours and the other left untreated. Subpopulations were then reanalyzed to determine whether they had retained a distribution consistent with the gating criteria used to obtain this narrow subpopulation and were susceptible to CHX treatment. We found that subpopulations R3 and R4 were susceptible to CHX treatment (Figure 3), whereas R5 and R6 did not change their fluorescence properties in response to CHX (data not shown). The fluorescence intensity of R2 was too low to detect after CHX treatment. The lack of susceptibility of the brighter R5-R6 subpopulations was most likely the result of their expressing predominantly stable proteins, which would be expected to provide more intense fluorescence.

We selected R4 for further screening in this study. We collected 10^6 ^cells from the shifted population, the left shoulder of the population observed in the CHX-treated but not in the untreated R4 cells (Figure 3). Plasmid DNAs were recovered from the sorted cells and were propagated in *Escherichia coli*, resulting in a total of 400 clones. The individual clones were stored in 15% glycerol LB medium in a 96-well format.

To perform second-round selection, we grouped the 400 clones into 12 pools, each composed of approximately 33 clones. The individual pools of clones were cultured and used for plasmid preparation. We transfected these 12 groups of plasmid DNA into 293T cells and again subjected them to FACS analysis and gating as before. The EGFP-C1 vector was used as a control. Because enhanced green fluorescent protein (EGFP) is a stable protein, its fluorescence intensity would not be changed by treatment with CHX. We found that eight of the 12 groups showed a decrease of the fluorescence intensity peak by 30-50% (compared to untreated cells) after 2 hours of CHX treatment. In four out of 12 groups, no change in fluorescence intensity was detected.

To isolate individual clones with the desired property, we randomly chose one of the eight CHX-responsive groups and characterized individual clones. We analyzed 30 clones from this group by individually transfecting them into 293T cells and determining the half-life by FACS-based analysis of CHX chase kinetics. We found out that 22 clones showed a decrease in fluorescence intensity ranging from 30 to 90% after treatment with CHX for 2 hours. Assuming first order kinetics of turnover, this single-time-point experiment implies that the proteins corresponding to these 22 clones have a range of half-lives ranging from about half an hour to 3-4 hours (Table 1). The 22 clones were partially sequenced and BLAST used to search for similar protein sequences in the National Center for Biotechnology Information (NCBI) public database. Of these, 19 corresponded to annotated genes in GenBank and the remaining three to unknown genes. Sequencing analysis also indicated that the inserts of these clones corresponded to full-length or near full-length translation reading frames.

**Figure 4:**
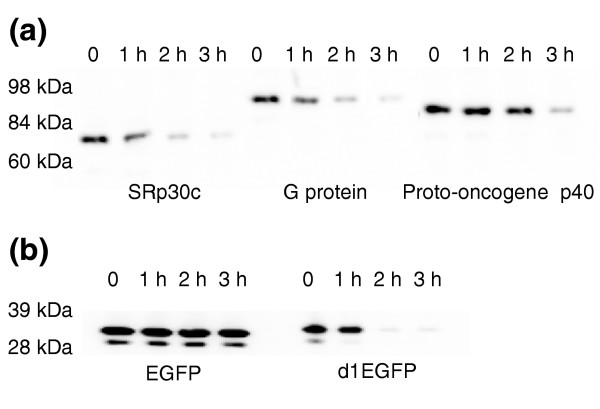
CHX chase analysis by western blot of three labile EGFP-fused library clones. **(a) **Cells were individually transfected with GFP-cDNA clones representing splicing factor SRp30c, guanine nucleotide-binding regulatory protein or cervical cancer proto-oncogene p40. Transfected cells were treated with CHX and were collected immediately thereafter or after 1, 2 or 3 hours for western blot analysis using anti-EGFP polyclonal antibody. The mobility of protein markers is indicated. **(b) **Cells were transfected with constructs expressing EGFP or d1EGFP, a destabilized form of GFP, and analyzed as in (a).

As no data are available on the intracellular turnover kinetics of the 19 identifiable proteins, we picked three clones - splicing factor SRp30c, a guanine nucleotide-binding regulatory protein (G protein), and cervical cancer 1 proto-oncogene protein - and examined their turnover by CHX chase and western blot analysis. These three clones (Table 1, numbers 5, 19 and 26) were estimated in the fluorescence-based screen to have diverse turnover kinetics; two of them have a half-life of less than 1 hour while the third turns over somewhat more slowly. To confirm these estimates of turnover by a means independent of GFP fluorescence, 293T cells were transfected with these clones, treated with CHX and periodically sampled over the next 3 hours. Western blot analysis of cell extracts with antibody to GFP showed that the abundance of all three fusion proteins diminished in the presence of CHX (Figure 4a). The half-life of the proteins determined by western blot analysis was similar to that determined by FACS analysis. Two of the proteins showed a half-life of about 1 hour, while the proto-oncogene protein appears to initiate abrupt degradation within about 2 hours of treatment with CHX. The results for all three proteins are thus consistent with those observed using the fluorescence-based screening method. As positive and negative controls, we similarly analyzed cells expressing a destabilized version of EGFP, d1EGFP, whose short half-life has been previously characterized [10], and a stable EGFP protein (Figure 4b).

**Figure 5:**
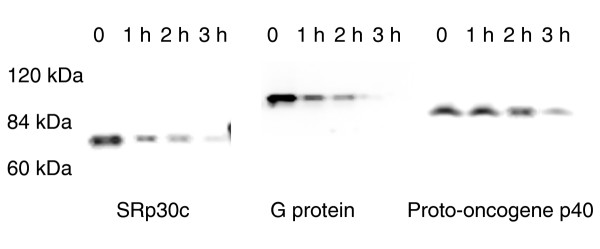
Cycloheximide chase analysis by western blot of three full-length myc-tagged cDNAs. Cells were transiently transfected to express splicing factor SRp30c, guanine nucleotide-binding regulatory protein or cervical cancer proto-oncogene p40, each with an amino-terminal myc epitope tag. Transfected cells were treated with CHX and samples subjected to western blot analysis using anti-myc antibody. The mobility of protein markers is indicated.

Sequencing analysis indicated that these three GFP fusion cDNAs do not contain a full-length coding sequence. SRp30c cDNA is missing 17 amino acids at its amino terminus, G protein 20 amino acids, and proto-oncogene p40 three amino acids. To exclude the possibility that the missing amino acids or the fused GFP domain contribute artifactually to protein liability, we amplified the full-length coding sequences of these three genes and expressed them as Myc fusion proteins. Their turnover was examined by CHX chase and western blot analysis with antibody to the Myc tag (Figure 5). Turnover rates assessed in this way were similar to those of the GFP fusion proteins obtained from library screening, ruling out the presence of these artifacts.

**Figure 6:**
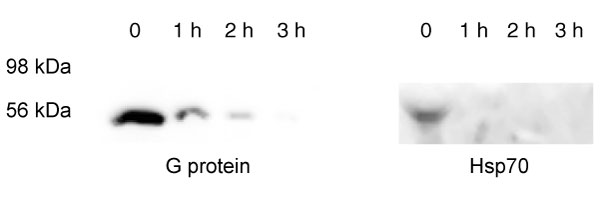
Cycloheximide chase analysis by western blot of two endogenous proteins. 293T cells were treated with CHX and samples subjected to western blot analysis using antibodies against G protein or Hsp70. The mobility of protein markers is indicated.

This technology is subject to two kinds of false-positive results. First, fusion to a detection tag such as GFP or Myc may affect the folding of tagged proteins, which could accelerate their turnover. Second, expression of the fusion proteins under the control of viral promoter elements could result in overexpression, with concomitant misfolding or failure to associate with endogenous interaction partners. To rule out these artifacts, we measured the degradation of native non-fusion endogenous counterparts of two of the proteins we identified, those for which antibodies were available. Turnover of the proteins associated with clone 19 and clone 25 was measured by CHX chase and western blot analysis. The results (Figure 6) demonstrated that the half-life of clone 19, a guanine nucleotide-binding regulatory protein (G protein), was less than 1 hour and the half-life of clone 25, heat-shock 70 kD protein (hsp70), was about 1 hour. The turnover of the native proteins is thus at least as fast as that of the corresponding clones analyzed in the screen, suggesting that the technology can accurately identify short-lived proteins.

## Discussion

The abundance of a given cellular protein is determined by the balance between its rate of synthesis and degradation. The two are of equal importance in their effect on the steady-state level. Furthermore, degradation determines the rate at which a new steady state is reached when protein synthesis changes [3]. Despite its importance, degradation, the 'missing dimension' in proteomics [7], has received far less comprehensive attention than synthesis. This deficiency has arisen because developing the tools for a proteome-wide study of protein turnover is technically challenging. Proteins that are labile tend to be present at low abundance, and methods for characterizing turnover time are laborious.

We have developed an efficient and rather specific screen by combining GFP fluorescence, as a high-throughput measure of protein abundance, with pharmacologic shutoff of protein synthesis. Of 30 clones that were recovered from the screen (Figure 1) and individually examined by CHX treatment and FACS analysis, 22 (73%) are associated with proteins with a half-life of less than 4 hours. Given the relative rarity of rapidly degraded proteins in the proteome [14], this result demonstrates the specificity of the screening method. We have so far analyzed a restricted subset of the clones that were recovered in our screening procedure - 30 clones present in one of eight positive pools (among 12) from the R4 population. A second population, R3, appears to be equally rich in clones responsive to CHX. Extrapolation from this small sample implies that perhaps 300-400 (that is, 22 × 8 × 2) clones within the GFP-cDNA library may be found to be associated with proteins that are labile according to our secondary screening criterion. In contrast to the results with the less bright R3 and R4 cell populations, the failure to detect a CHX-sensitive subpopulation among the brighter R5-R6 cells is consistent with the expectation that labile proteins tend to be of lower abundance than more stable proteins.

For some of the proteins uncovered in this survey, rapid turnover can be rationalized as intrinsic to their cellular function. SRp30c factor (accession number U87279) is responsible for pre-mRNA splicing. Alterative splicing is a commonly used mechanism to create protein isoforms. It has been proposed that organisms regulate alternative splice site selection by changing the concentration and activity of splicing regulatory proteins such as SRp30c in response to external stimuli [15]. The finding that SRp30c is a short-lived protein is consistent with its postulated regulatory function.

The G proteins are a ubiquitous family of proteins that transduce information across the plasma membrane, coupling receptors to various effectors [16,17]. About 80% of all known hormones, neurotransmitters and neuromodulators are estimated to exert their cellular regulation through G proteins. The G protein (accession number M69013) shown here to short-lived is a G protein α subunit that transduces signals via a pertussis toxin-insensitive mechanism [18]. Like other pertussis toxin-insensitive G proteins such as the Ga12 class, it causes the activation of several cytoplasmic protein tyrosine kinases: Src, Pyk2 (proline-rich tyrosine kinase 2) and Fak (focal adhesion kinase) [19]. However, it is not known how this G protein is regulated. Its rapid turnover suggests a testable mechanism of its regulatory activation. Cervical cancer 1 proto-oncogene protein p40 (accession number AF195651), is a third protein shown here to turn over rapidly, but its function is unknown. Further studies of its turnover may provide important information on its function and regulation.

In mammalian cells, proteasomes have the predominant role in the degradation of short lived proteins, whereas lysosomal degradation appears to be quantitatively less important [20]. Determining the mechanism that cells use to degrade the proteins uncovered by the method described here will require the use of specific inhibitors [21]. Before degradation, most short-lived proteins are covalently coupled to multiple copies of the 76-amino-acid protein ubiquitin [22], a reaction catalyzed by a series of enzymes [23]. These ubiquitinated proteins are recognized by the 26S proteasome and degraded within its hollow interior [24]. This system of regulated degradation is central to such processes as cell-cycle progression, gene transcription and antigen processing. A few proteins have been found to be exceptions [25,26]; like ODC, they do not require ubiquitin modification for degradation by the proteasome. In most cases it is not clear how short-lived proteins are selected to be modified and degraded. Some rapidly degraded proteins have been shown to contain an identifiable 'degradation domain'. Removal of this degradation domain makes such proteins stable, and appending this domain to a stable protein reduces its stability. Such a degradation domain has been identified in a number of short-lived proteins, including the carboxy terminus of mouse ODC [6,27] and the destruction box of cyclins [28]. In some cases, the signal is a primary sequence - like the PEST sequence [29,30]. However, the identifiable structural features of such degradation domains are not sufficiently uniform to provide a reliable guide to identifying labile proteins. The method we have described does not use ubiquitin conjugation as a search criterion. This approach thus has the potential to discover labile proteins regardless of whether ubiquitin modification plays a role in their turnover. Once a large and representative sample of short-lived proteins is identified, a search for structural motifs among these proteins may facilitate the discovery of those motifs which correlate to protein degradation.

## Conclusions

In this study we have developed an innovative technology to identify labile proteins using GFP-fusion expression libraries. Using this technology we have discovered short-lived proteins in a high-throughput format. This technology will greatly facilitate the discovery and study of short-lived proteins and their cellular regulation.

## Materials and methods

### Construction of GFP-cDNA expression libraries

Messenger RNAs from brain, liver, and the HeLa cell line (Clontech) were used as templates for cDNA synthesis, using a cDNA synthesis kit from Stratagene according to the manufacturer's recommendation, with some modifications. First-strand cDNA was synthesized using an oligo(dT) primer-linker containing an *Xho*I restriction site and with StrataScript reverse transcriptase. Synthesis was performed in the presence of 5-methyl dCTP, resulting in hemimethylated cDNA, which prevents endogenous cutting within the cDNA during cloning. Second-strand cDNA was synthesized using *E. coli *DNA polymerase and RNase H. Adaptors containing *Eco*RI cohesive ends were introduced into the double-stranded cDNA, which were then digested with *Xho*I. The cDNAs contained two different sticky ends: 5' *Eco*RI and 3' *Xho*I. The cDNAs were separated on a 1% SeaPlaque GTG agarose gel in order to collect those larger than 800 bp. After extracting cDNAs from the agarose gel with AgarACE-agarose-digesting enzyme followed by ethanol precipitation, the cDNAs were directionally cloned into EGFP-C1/2/3 expression vectors with three open reading frames (ORFs) (Clontech). The vectors were modified within the multiple cloning sites in order to be compatible with the cDNA orientation. By this means, cDNA ORFs were aligned to the carboxy terminus of EGFP. The host cell used for plasmid transfection and expression, 293T, expresses the SV40 large T antigen. Therefore, the cDNA EGFP-C1/2/3 vector containing the SV40 origin of replication can replicate independently from chromosome DNA in the host cells, which facilitates the recovery of plasmid DNAs from the host cells.

### Transfection of the libraries into 293T cells

293T cells were cultured at 37°C in DMEM (Invitrogen) supplemented with 10% FBS, 1% nonessential amino acids and 100 U/ml penicillin, 0.1 mg/ml streptomycin. One day before transfection, cells were seeded in 10-cm plate in 10 ml growth medium without antibiotics. Transfection was performed using Lipofectamine 2000 reagent according to the manufacturer's instructions. Samples (25 μg) of a cDNA library were diluted in 1.5 ml Opti-MEM (Invitrogen). Lipofectamine 2000 was diluted in 1.5 ml Opti-MEM and mixed with diluted DNA. After 20 min incubation, the DNA-Lipofectamine 2000 complex was added to the cells. The cells were incubated for 16 h before analysis.

### FACS analysis of GFP-expressing cells

Cells were harvested by trypsinization, washed, and resuspended in DMEM. Cytometric analysis and sorting were performed using a hybrid cell sorter combining a Becton Dickinson FACStarPLUS optical bench with Cytomation Moflo electronics (Stanford Beckman Center shared facility). Green fluorescence was measured using a 525/50 band pass filter. Gates were set to exclude cellular debris and the fluorescence intensity of events within the gated regions was quantified. Fluorescence-activated cell sorting was performed with a lower forward scatter threshold to detect transfected cells while ensuring that debris and electronic noise were not captured as legitimate events. Transfection efficiency was so high that normal voltages for detecting GFP were reduced. For fractionation, the cell population was gated on the basis of the fluorescence intensity. Cells were sorted at a rate of 8,000 events/sec. 10^6 ^cells were collected in 12 × 75 mm glass tubes containing 200 μl serum to enhance the cell survival rate. For short-lived protein screening, sorted cells were recultured in a 12-well plate and treated with or without 100 μg/ml CHX for 2 h. The cells then were collected and subjected to FACS analysis and sorting. The cells showing a decrease in fluorescence intensity with CHX treatment were collected for further analysis.

### Plasmid recovery

Plasmid DNA was extracted from sorted cells using a Qiagen mini-plasmid preparation kit. Plasmid DNAs were eluted in water and transformed into electro-competent DH10B *E. coli* (Invitrogen). Bacterial colonies were transferred to 96-well plates containing LB with 50 μg/ml kanamycin and 30% glycerol. After overnight growth at 37°C, the colonies are stored at -80°C. Plasmid DNAs were prepared from individual clones, sequenced and BLAST searches performed against the NCBI database.

### Construction of Myc-tagged full-length coding sequences of genes

To obtain full-length coding sequence of the genes, we amplified them with a human full-length cDNA kit (Panomics) according to the manufacturer's instructions. The full-length coding sequences of cDNAs were then cloned into the pCMV-Myc vector (Clontech) for expression in 293T cells.

### Western blot analysis of protein degradation

The plasmid DNAs of individual clones were prepared and transfected into 293T cells. The transfected cells, with or without CHX treatment, were collected in PBS and cell lysates were prepared by sonication. Proteins were resolved by SDS-polyacrylamide gel electrophoresis and transferred to a membrane. Fusion proteins were detected using a polyclonal antibody against GFP (Clontech), a monoclonal antibody against the Myc epitope (Sigma), a polyclonal antibody against G protein (Santa Cruz) or an antibody against Hsp70 (Santa Cruz). Bands were visualized with SuperSignal West Pico kit (Pierce).

## Additional data files

Additional data file 1 contains the original data used to perform this analysis and is available with the online version of this paper.

## Supplementary Material

Additional data file 1 The original data used to perform this analysis Click here for additional data file

## Figures and Tables

**Table 1:** The estimated half-lives of 22 labile proteins

Clone	Accession number	Gene description	Stability	Estimated half-life (h)
2	BC005843	Similar to SH3-containing protein	Short-lived	2
3	AF176555	A-kinase anchoring protein	Short-lived	3
4	AF209502	Calpain	Short-lived	1
5	U87277	Splicing factor SRp30c	Short-lived	0.5
9	BC000804	Adaptor-related protein complex	Short-lived	2
10	BC011384	ATP synthase, H^+ ^transporting	Short-lived	2
12	BC005380	Apolipoprotein A-I	Short-lived	2
14	BC007513	H19, imprinted maternally expressed	Short-lived	2
18	AP000014	Function unknown	Short-lived	2
19	M69013	Guanine-nucleotide-binding regulatory protein	Short-lived	0.5
21	BC003128	CGI-89 protein	Short-lived	2
22	AL109795	Function unknown	Short-lived	2
23	X89399	Ins(1,3,4,5)P_4_-binding protein	Short-lived	2
25	BC057397	Heat shock 70 kDa protein 1A	Short-lived	2
26	NM_015416	Cervical cancer 1 proto-oncogene protein p40	Short-lived	2
27	AF248272	Gag-Pro-Pol precursor protein gene.	Short-lived	3
28	X07868	Insulin-like growth factors II	Short-lived	2
29	NM_000062	Serine (or cysteine) proteinase inhibitor	Short-lived	2
30	AF068706	Gamma2-adaptin (G2AD)	Short-lived	2
32	AK054590	Function unknown	Short-lived	3
33	BC000404	Thyroid hormone receptor interactors 13	Short-lived	2

The clones were recovered as described in the text and their half-lives were estimated by FACS-based analysis of CHX chase kinetics. All 22 clones were partially sequenced and BLAST analysis performed to identify similar protein sequences in the NCBI public database.
